# A Multiparametric and High-Throughput Platform for Host–Virus Binding Screens

**DOI:** 10.1021/acs.nanolett.2c04884

**Published:** 2023-03-09

**Authors:** Jan Schlegel, Bartlomiej Porebski, Luca Andronico, Leo Hanke, Steven Edwards, Hjalmar Brismar, Ben Murrell, Gerald M. McInerney, Oscar Fernandez-Capetillo, Erdinc Sezgin

**Affiliations:** † Science for Life Laboratory, Department of Women’s and Children’s Health, Karolinska Institutet, 17165 Solna, Sweden; ‡ Science for Life Laboratory, Division of Genome Biology, Department of Medical Biochemistry and Biophysics, Karolinska Institutet, 17165 Stockholm, Sweden; § Department of Microbiology, Tumor and Cell Biology, Karolinska Institutet, 17165 Stockholm, Sweden; ∥ Science for Life Laboratory, Department of Applied Physics, Royal Institute of Technology, 17165 Solna, Sweden; ⊥ Genomic Instability Group, Spanish National Cancer Research Centre (CNIO), Madrid 28029, Spain

**Keywords:** silica beads, virus binding, lipid bilayer, ACE2, neuropilin-1, flow cytometry

## Abstract

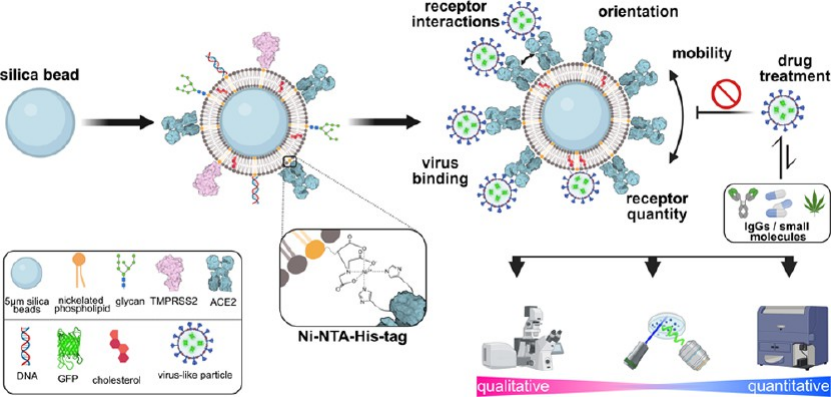

Speed is key during infectious disease outbreaks. It is essential, for example, to identify critical host binding factors to pathogens as fast as possible. The complexity of host plasma membrane is often a limiting factor hindering fast and accurate determination of host binding factors as well as high-throughput screening for neutralizing antimicrobial drug targets. Here, we describe a multiparametric and high-throughput platform tackling this bottleneck and enabling fast screens for host binding factors as well as new antiviral drug targets. The sensitivity and robustness of our platform were validated by blocking SARS-CoV-2 particles with nanobodies and IgGs from human serum samples.

Emerging microbial pathogens, such as bacteria, fungi, and viruses, tremendously challenge human health and cause significant economical and societal burden worldwide. Therefore, tools facilitating and improving pandemic preparedness are of uttermost importance to minimize these negative effects. Current state-of-the-art methods, such as enzyme-linked immunosorbent assay (ELISA), reverse transcription-polymerase chain reaction (RT-PCR), and RT loop-mediated isothermal amplification (RT-LAMP) usually rely on bulk measurements resulting in a single readout-value.^1^ In addition, during the peaks of SARS-CoV-2 pandemic, RT-PCR instruments were used to capacity slowing down pandemic surveillance and highlighting the need for additional readout-systems. Especially flow cytometry, enabling fast and high-throughput measurements of complex mixtures, is widely used in clinics for immunophenotyping and would be an attractive and broadly available technique for such purposes.^2^ For example, previous work showed that combining flow cytometry with Jurkat T-cells stably expressing SARS-CoV-2 Spike can be a sensitive tool to detect neutralizing antibodies in human serum samples.^3^

To complement existing bulk measurement methods, we aimed to develop a fast and high-throughput platform to study host–pathogen interactions. The system reconstitutes host cell proteins as well as lipid bilayer, which is mostly neglected in current molecular interaction methods but often hosts important attachment factors. The complexity of the mammalian plasma membrane consisting of thousands of different lipids and proteins embedded between an outer glycocalyx and inner cortical cytoskeleton is overwhelming. This complexity hampers our efforts for the fast identification of important interaction partners. To overcome this bottleneck and reduce complexity, bottom-up model membrane systems are attractive alternatives which allow for precise control over composition and properties. Among these, planar supported lipid bilayer systems (SLBs) were widely used^4^ but do not account for cells’ three-dimensional nature. Three-dimensional model systems such as large unilamellar vesicles (LUVs), giant unilamellar vesicles (GUVs), and cell-derived giant plasma membrane vesicles (GPMVs) help to recreate cellular curvature but are challenging to use in high-throughput flow cytometry because of their fragility and size-inhomogeneity.

For this reason, we coated cell-sized 5 μm silica beads with a lipid bilayer consisting of 98 mol % 1-palmitoyl-2-oleoyl-glycero-3-phosphocholine (POPC) doped with 2 mol % of a nickelated anchoring lipid (18:1 DGS-NTA(Ni)). Next, we attached His-tagged host-cell proteins of interest (via NTA(Ni)-His coupling) to membrane-coated beads to generate functionalized bead-supported lipid bilayers (fBSLBs) serving as minimal synthetic host-cells (Figure 1A). In contrast to methods relying on random surface-adsorption, fBSLBs ensure proper protein orientation, tightly controllable receptor mobility and density as well as molecular interactions at the membrane plane. In addition, the presence of a hydrophobic lipid bilayer more closely mimics the cellular environment and enables discrimination between binding preferences of pathogens to either host-cell proteins or lipids. For example, surface proteins of several viruses can bind different host-cell lipids facilitating cellular uptake and shaping viral tropism.^5^ In this study, we show that fBSLBs carrying different host cell receptors, such as angiotensin-converting enzyme 2 (ACE2), can serve as a highly diverse platform to screen for molecules influencing host–pathogen interactions and the blocking efficiency of neutralizing antibodies present in human serum samples. Its fast implementation, easy adaptability of multiple parameters, and high-throughput capability propel our method as an important platform to study host–pathogen interactions.

**Figure 1:**
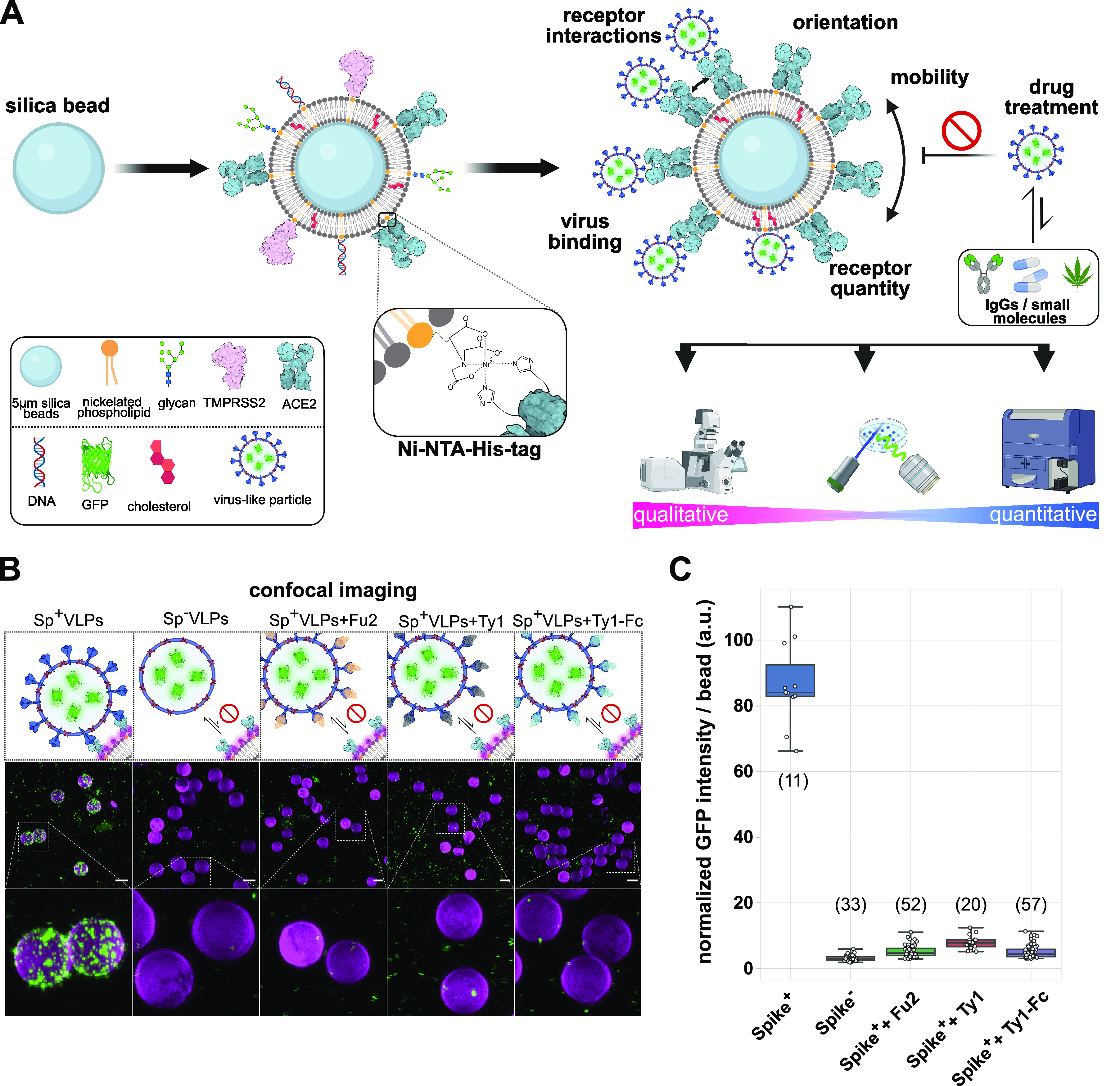
Design and characterization of multiparametric and high-throughput platform based on fBSLBs to study host–virus interactions. (A) Scheme depicting the bottom-up assembly of fBSLBs and available readout techniques. (B) LSM maximum-intensity projections of fBSLBs (magenta, lipid dye ASR-PE) and VLPs (green, EGFP) showing specific interaction between SARS-CoV-2 Spike VLPs (Sp^+^VLPs) and ACE2-fBSLBs. (C) Quantification of viral GFP-signal per fBSLBs of each condition from panel B shows specific attachment of Sp^+^VLPs to ACE2-fBSLBs (median = 84.05, *N* = 11) and no interaction between Sp^–^VLPs and ACE2-BSLBs (median = 2.88, *N* = 33) and nanobody-pretreated Sp^+^VLPs and ACE2-BSLBs (Fu2, median = 4.70, *N* = 52; Ty1, median = 7.77, *N* = 20; Ty-Fc, median = 4.41, *N* = 57). Graph shows representative data of three independent experiments. Boxplot with overlay of individual data points, median as black center line, box showing the quartiles and whiskers from minimum to maximum value. Illustrations were created using Biorender.com and Inkscape.

Upon coating of 5 μm silica beads with POPC:DGS-NTA(Ni) 98:2 mol % of liposome solution, we first verified proper bilayer formation by measuring diffusion of a fluorescent lipid analogue using fluorescence correlation spectroscopy (FCS) (Figure S1), which matched with previous data.^6,7^ We generated fBSLBs (labeled with fluorescent lipid dye: Abberior STAR RED 1,2-dioleoyl-*sn*-glycero-3-phosphoethanolamine lipid (ASR-PE)) carrying ACE2 and studied their interaction with SARS-CoV-2 Spike expressing virus-like particles (*Sp*^*+*^*VLPs*, carrying EGFP) using confocal microscopy (Figure 1B,C). To quantify VLP-binding per bead, we developed an automated image analysis workflow using Fiji^8^ (Figure S2). While there was strong interaction between ACE2-fBSLBs and Sp^+^VLPs, no interaction was observed with VLPs with no Spike (*Sp*^*–*^*VLPs*) and Sp^+^VLPs pretreated with SARS-CoV-2 neutralizing Spike nanobodies which were shown to be potent tools to neutralize SARS-CoV-2 by blocking the interaction between Spike receptor-binding domain (RBD) and its host receptor ACE2.^9,10^ Thus, fBSLBs can serve as powerful screening platform to identify efficient inhibitors with therapeutic potential.

To increase the number of data points and decrease acquisition time, we performed fast, quantitative, 3D lattice light-sheet microscopy (LLSM) and quantified viral loads per fBSLB (Figure 2A,B) which confirmed confocal microscopy data. To screen several tens of thousands of fBSLBs within minutes, fast and high-throughput flow cytometry can be used due to the firm nature of fBSLBs. Individual fBSLBs were easily detected by their specific scattering signal and presence of the lipid bilayer confirmed by ASR-PE labeling, while VLPs are tagged with EGFP. Upon addition of ASR-PE and Sp^+^VLPs to ACE2-fBSLBs, we observed a strong increase of fluorescence intensity per bead both in virus (green) and in membrane (red) channels (Figure 2C). Moreover, the virus signal decreased significantly upon nanobody treatment, confirming the neutralizing ability of nanobodies. Hence, fBSLBs enable the study of host–virus interactions using quantitative high-throughput flow cytometry which is usually not feasible due to the small size of viral particles. Moreover, it serves as a powerful platform to study concentration-dependent effects of molecules on the binding between viruses and host-cell receptors. To show this, we determined optimal concentrations of ACE2 on the fBSLBs and the amount of Sp^+^VLPs by titration series (Figure S3).

**Figure 2:**
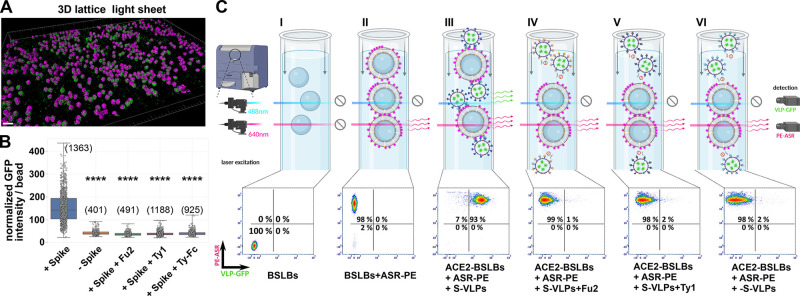
High-throughput measurements using fBSLBs. (A) Fast and quantitative LLSM enabling big-volume renderings of ACE2-fBSLBs (magenta, lipid dye ASR-PE) interacting with Sp^+^VLPs (green, EGFP). (B) Quantification of VLP-GFP signal per bead proves specific interaction between Sp^+^VLPs and ACE2-fBSLBs (*N* > 400). Boxplot with overlay of individual data points, median as black center line, box showing the quartiles and whiskers from minimum to maximum value. Sp^+^VLPs show significant increased binding to ACE2-fBSLBs as compared to the other groups (*p* < 0.0001). (C) Fast high-throughput screening of interaction between VLPs and ACE2-fBSLBs using flow cytometry. Strong signal of the fluorescent lipid ASR-PE (*y*-axis) confirms functional bilayer formation, and the interaction of VLPs with fBSLBs can be followed by intensity changes in the VLP-GFP channel (*x*-axis) (*N* > 8500 per condition). Graphs show representative data of three independent experiments. Illustrations were created using Biorender.com and Inkscape.

Besides ACE2, other receptors have been described to contribute to SARS-CoV-2 binding to the host-cell surface and subsequent infection. For this reason, we tested interaction of Sp^+^VLPs with reported host-cell binding partners neuropilin-1 (CD304),^11,12^ basigin (CD147),^13^ DPP4 (CD26),^14,15^ and TMPRSS2^16,17^ using fBSLBs in combination with flow cytometry (Figures 3, S4, and S5). As expected, Sp^+^VLPs showed strongest interaction with ACE2-fBSLBs (Figure 3A). Interestingly, Sp^+^VLPs also interacted moderately with CD304-fBSLBs and slightly with TMPRSS2-fBSLBs, confirming that these two proteins might act as host binding factors, but neither interaction was as strong as of ACE2-fBSLBs. No binding was observed for CD147-fBSLBs or CD26-fBSLBs, suggesting that these proteins cannot act as host binding factors alone and might require additional host-cell binding elements. Notably, binding of CD304-fBSLBs to VLPs was independent of Spike-protein on their surface. Fu2 treatment that neutralizes Spike-ACE2 interaction did not show any effect on Sp^+^VLPs binding to CD304-fBSLBs (Figure 3A). Moreover, VLPs without Spike (Sp^–^VLPs) also bound to CD304-fBSLBs effectively (and TMPRSS2-fBSLBs slightly) while they did not bind the other proteins we tested (Figure 3B). This suggests the presence of other interaction partners on the viral particles to these proteins. To check whether Spike-independent binding plays a role even in the presence of Spike-ACE2 interaction, we performed dual-receptor screens with equimolar concentrations of ACE2 and CD304 or TMPRSS2 (Figure 3C). Upon blocking of the Spike-ACE2 interaction by incubation of Sp^+^VLPs with Fu2 nanobody, there was still residual binding on ACE2+CD304-fBSLBs (Figure 3C). These results show that in the presence of equimolar amounts of both receptors, ACE2 mediates stronger binding but CD304-mediated binding is not negligible.

**Figure 3:**
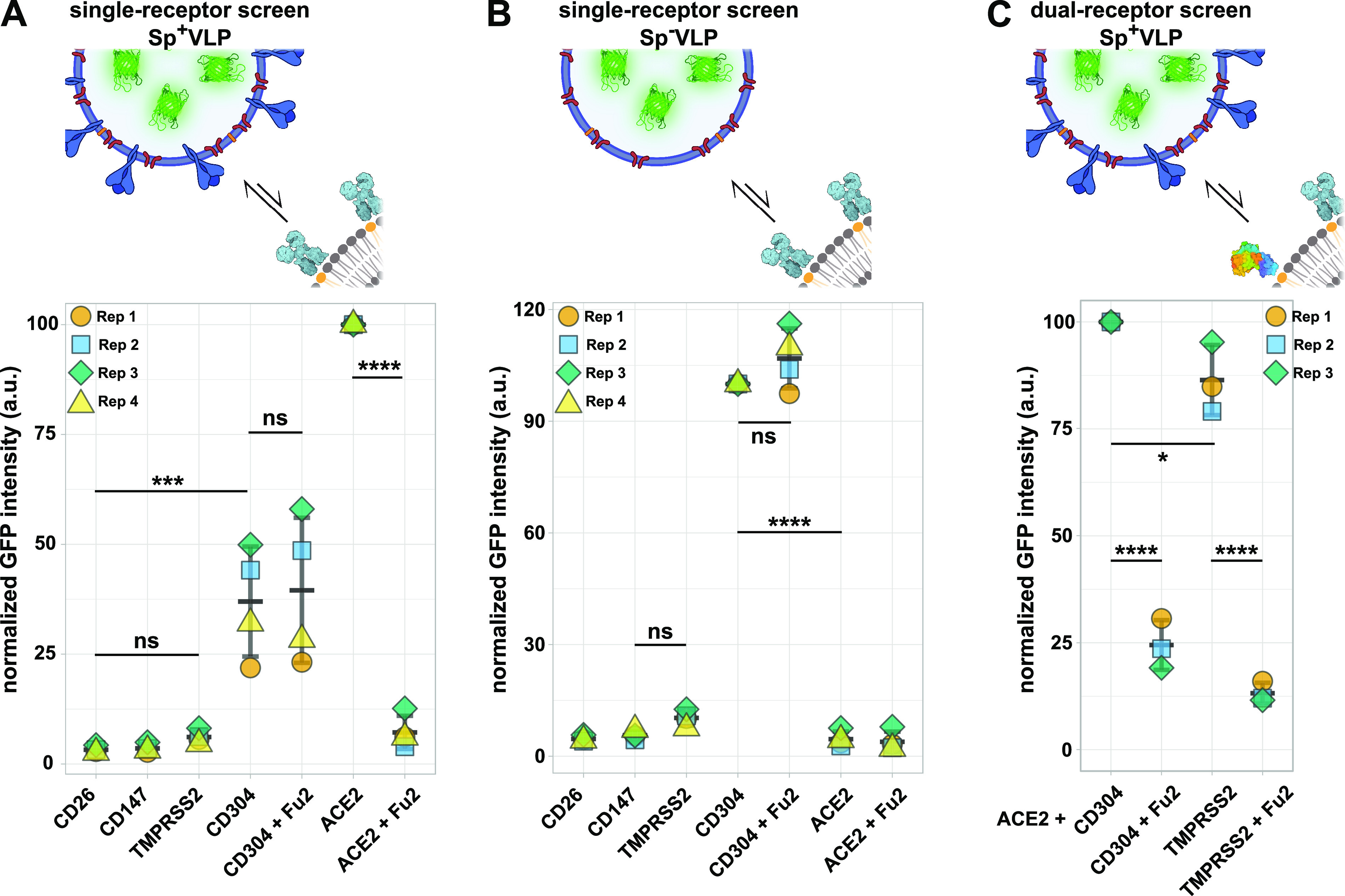
Receptor screening using fBSLBs. (A) Application of fBSLBs to study interaction between VLPs and different host-binding partners using flow cytometry. Scheme depicting fBSLBs with a single protein interacting with Sp^+^VLPs. Plot shows the ACE2-normalized medians of four different biological replicates (marked by different colors and shapes) from populations with ≈10 000 data points. The error bars show the standard deviation. Besides ACE2 (mean, 100), specific but less pronounced binding was also observed for CD304 (mean, 37), even when Sp^+^VLPs were blocked with Fu2 nanobody (mean, 40). (B) Scheme depicting fBSLBs with single protein interacting with Sp^–^VLPs. Plot shows the CD304-normalized medians of four different biological replicates (marked by different colors and shapes) from populations with ≈10 000 data points. Despite the absence of Spike, strong binding to CD304-fBSLBs was observed, even when Sp^–^VLPs were pretreated with Fu2 nanobody. (C) Dual receptor screen using fBSLBs and flow cytometry. Scheme depicting fBSLB coated with two proteins interacting with Sp^+^VLPs. BSLBs were functionalized simultaneously with equimolar concentrations of ACE2 and CD304 or TMPRSS2. Plot shows the ACE2+CD304-normalized medians of three different biological replicates (marked by different colors and shapes) from populations with ≈7000 data points. Despite blocking the interaction between Sp^+^VLPs and ACE2 with Fu2, there was still residual binding due to CD304 (mean, 24).

fBSLBs allow tight control on the composition of not only surface proteins but also lipids. We made use of this and screened for reported lipid co-receptors for Spike, such as GM1 gangliosides.^18^ Despite varying GM1 concentrations in fBSLBs, we could not observe any concentration-dependent binding of VLPs pseudotyped with Spike, beta-Spike, delta-Spike, Ebola virus glycoprotein (GP) or without any viral protein (Figure S6). These results highlight the need for additional high-affinity host-cell binding factors for efficient virus–host interaction.

Key for pandemic containment is surveillance of convalescent serum samples and their ability to block the interaction between virus and host cell receptors. Virus-specific antibody levels in human serum are usually proportional to neutralization of the virus and can be used to predict disease-outcome or the need for additional booster vaccinations.^19,20^ Moreover, it is very important to understand whether antiviral IgGs in prevalent serum samples still protect from upcoming new variants to decide for vaccine-adjustments and therapeutic treatment options. To show the potential of our method to answer these questions, we first determined the amount of Spike-IgGs in three human serum samples using a bead-based assay in combination with flow cytometry (Figure 4A). Glass beads were coated with recombinant Spike receptor binding domain (RBD), incubated with serum samples, and anti-Spike IgGs were detected by labeling with secondary dye-conjugated anti-human antibodies. After we determined the relative levels of anti-Spike IgGs in the three serum samples, we blocked Sp^+^VLPs with the different serum samples and studied the interaction with ACE2-fBSLBs. The amount of anti-Spike IgGs perfectly correlated with the blocking efficiency, highlighting the ability of this method as a powerful tool for pandemic surveillance (Figure 4B).

**Figure 4:**
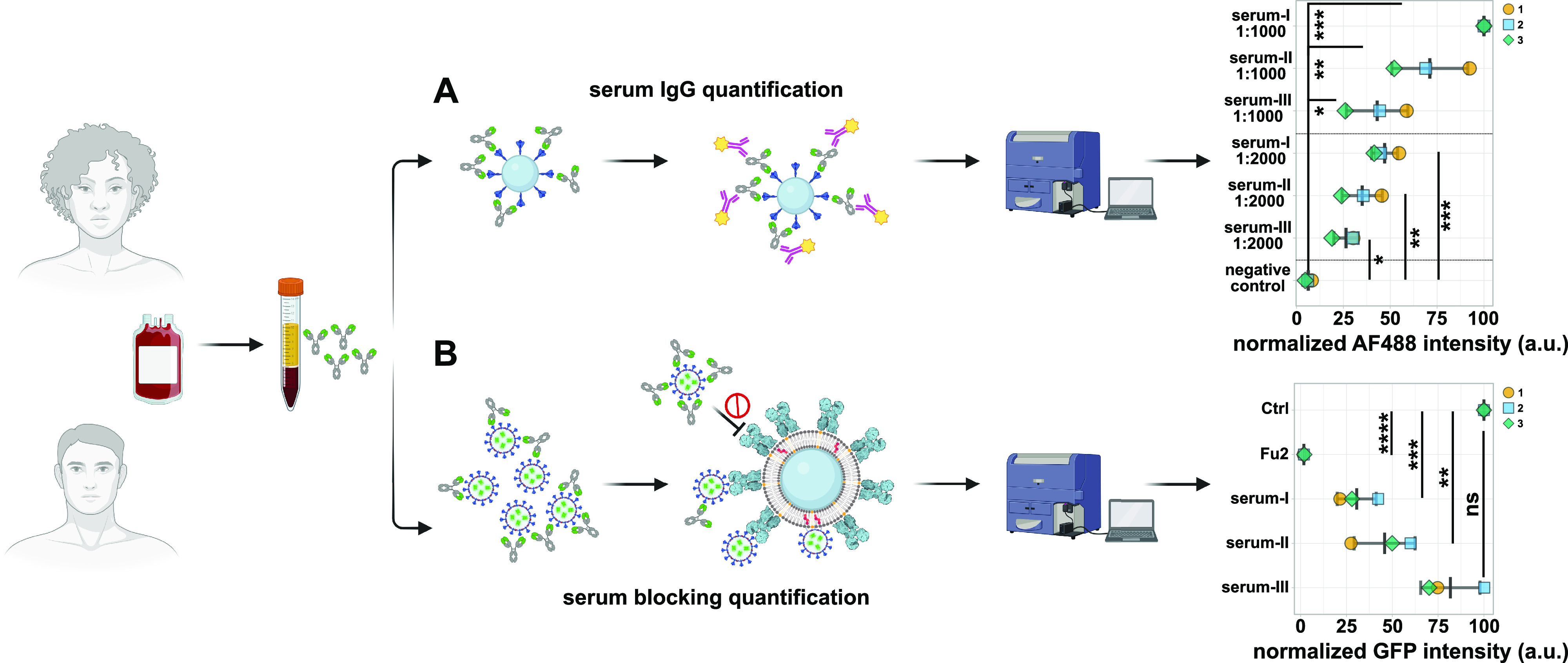
Application of fBSLBs to study blocking efficacy of neutralizing antibodies in human blood serum samples. Scheme showing the processing of human serum samples to quantify amount of anti-Spike IgGs and their capacity to block the interaction between Sp^+^VLPs and ACE2-fBSLBs. (A) Plot illustrates the serum-I-normalized medians of three different biological replicates to quantify the amount of anti-Spike IgGs in three different serum samples at different dilutions. (B) Plot shows the positive-control-normalized medians of three different biological replicates to quantify the blocking efficiency between Sp^+^VLPs and ACE2-BSLBs for the three different serum samples in panel A. The amount of anti-Spike-IgGs in the serum samples correlates with blocking efficiency. Illustrations were created using Biorender.com and Inkscape.

Quick response to pandemic outbreaks is of uttermost importance for disease and damage control. Our platform relies on material and molecules which are available from early pandemic onset, such as the sequence of viral structural proteins and potential interaction partners. Exploiting highly specific Ni-NTA-His-tag conjugation makes the platform highly versatile and accessible. While our method requires his-tagged proteins which could be time-consuming to produce, this chemistry is widely used for protein purification and His-tagged proteins are available from a myriad of commercial resources. Screening of potential host-cell receptors and co-receptors, including lipids, can be done within a few hours using qualitative and high-throughput quantitative readout platforms. In contrast to other methods, our method enables tight control of multiple parameters such as lipid composition, receptor mobility, receptor orientation, receptor–receptor interactions, and local receptor densities. The platform allows determination of serum–virus neutralization capacity in a safe laboratory environment within hours. Moreover, the presence of lipid bilayer more closely mimics the cellular environment and can help to entangle the complex interplay between virus–receptor and virus–bilayer interactions which are often difficult to discriminate. Due to its highly defined bottom-up assembly, fBSLBs are not prone to cellular heterogeneity, e.g., due to differences in cell-cycle states, transcription, and translation, which can complicate drug screens. However, this cellular heterogeneity could fine-tune host–pathogen interactions which is challenging to reproduce with our platform. Recent advances on coating beads with native cellular membranes would be an opportunity to recreate this complexity,^21,22^ yet it would compromise from controlled composition. Another limitation of fBSLBs is its inability to initially detect cellular toxic compounds. This can also be advantageous since substances showing both cellular toxicity and binding inhibition are identified with our platform and not directly discarded. Further efforts in reducing cellular toxicity while maintaining inhibitory effects of these molecules in cell-based screens would be an exciting way to find new drug targets.

Our broadly accessible platform enables us to perform fast and high-throughput drug screens and to discriminate whether drugs act on the virus particles or on the host-cell receptors. Due to its bottom-up design, our method should be readily extensible to other biomolecules (e.g., glycocalyx, DNA, RNA) and pathogens (bacteria, fungi) making it a valuable tool for future pandemic preparedness.

## Supporting Information Available

Materials and methods section; Figure S1 showing proof of functional lipid-bilayer formation around 5 μm silica beads; Figure S2 showing automated image analysis workflow to determine VLP-GFP signal per fBSLB; Figure S3 showing optimal protein- and VLP-concentrations determined by titration experiments; Figure S4 showing flow cytometry gating strategy; Figure S5 showing median-normalized raw data distributions of single- and dual-receptor screens; Figure S6 showing GM1-fBSLBs binding to VLPs with different viral proteins (PDF)

## Notes

Raw data will be available upon publication (FigShare DOI: 10.17044/scilifelab.20517336).
