# Supplementary material for: A Multiparametric and High-Throughput Platform for Host–Virus Binding Screens
Source: Nano Lett. 2023 Mar 9;23(9):3701–7. doi: 10.1021/acs.nanolett.2c04884 (PMC10176574; doi:10.1021/acs.nanolett.2c04884)
Supplement: Supplementary file 1 — nl2c04884_si_001.pdf [file nl2c04884_si_001.pdf]

A Multiparametric and High-Throughput Platform for Host-Virus Binding Screens

Jan Schlegel[1], Bartlomiej Porebski[2], Luca Andronico[1], Leo Hanke[3], Steven Edwards[4], Hjalmar Brismar[1,4], Ben Murrell[3], Gerald M. McInerney[3], Oscar Fernandez-Capetillo[2,5], Erdinc Sezgin[1]*

[1] Science for Life Laboratory, Department of Women's and Children's Health, Karolinska Institutet, 17165, Solna, Sweden.

[2] Science for Life Laboratory, Division of Genome Biology, Department of Medical Biochemistry and Biophysics, Karolinska Institutet, 17165, Stockholm, Sweden.

[3] Department of Microbiology, Tumor and Cell Biology, Karolinska Institutet, 17165, Stockholm, Sweden.

[4] Science for Life Laboratory, Department of Applied Physics, Royal Institute of Technology, 17165, Solna, Sweden.

[5] Genomic Instability Group, Spanish National Cancer Research Centre (CNIO), Madrid, 28029, Spain.

* Corresponding author:
Erdinc Sezgin
Email: erdinc.sezgin@ki.se

Materials and Methods

fBSLB preparation

fBSLBs were prepared similar as described previously [1]. For preparation of one batch of fBSLBs, 1x10^7 5µm silica beads (Bangs Laboratories) were vortexed thoroughly and washed three times with PBS using 1500xg and 30 seconds centrifugation steps. Beads were coated with lipid bilayers of defined compositions by incubation with 100µl 0.5mg/ml liposomes shaking at 1400rpm for 30 minutes. Liposomes were formed by mixing lipids dissolved in chloroform, solvent evaporation under a steam of nitrogen, re-hydration, and tip-sonication (Branson Sonifier 250). To prepare fBSLBs with His-tagged proteins, a lipid mixture consisting of 98mol% 16:0-18:1 POPC and 2mol% 18:1 DGS-Ni:NTA (Avanti Polar Lipids) was used. After bilayer formation beads were washed two more times with PBS and 15pmol of His-tagged proteins added (Sino Biological: ACE2-His (10108-H08H); Neuropilin-1-His (CD304,10011-H08H), CD147-His (10186-H08H), CD26-His (10688-H08H), LSBio: TMPRSS2-His (LS-G15055-50)). For dual receptor screens 5pmol of each individual receptor were applied simultaneously. After 20 minutes on a rotary shaker the bilayer of fBSLBs was optionally directly labelled with a fluorescent lipid analogue followed by 2 washing steps with PBS. Final fBSLBs were diluted in 500µl PBS and used the same day. To study host-virus interactions, 20µl of fBSLBs were mixed with 15µl of GFP-tagged pseudotyped VLPs and incubated for 30 minutes on a rotary shaker at room temperature and directly used for microscopy or flow cytometry. Optionally, VLPs were pre-treated for 20 minutes on ice with 2µM Ty1, Ty1-Fc or Fu2 nanobodies [2,3]. Assuming spherical shape of the 5µm silica beads, a bilayer thickness of 6nm, equal anchoring lipid distribution between inner and outer leaflet of 2mol% 18:1 DGS-NTA(Ni) and an area of 0.71nm^2 for each lipid headgroup we approximate the theoretical number of accessible anchoring lipids per bead to be ~ 2 217 702. Since we used poly-histidine tagged proteins with 10-12 histidine per protein, one his-tagged protein will bind to 5-6 anchoring lipids and result in ~406 579 proteins per bead. For an individual experiment ~4.07x10^{12} his-tagged proteins (~6.7pmol) should be sufficient to saturate all lipid binding sites on the beads which is below the amounts used for individual receptor (15pmol) and dual receptor (2x5=10pmol) screens.

VLP preparation

Mycoplasma-free HEK293T cells were cultured in DMEM supplemented with 10% FCS and grown to ~70% confluency in T75 cell culture flasks. To produce VLPs, cells were co-transfected using Lipofectamine 3000 and 15µg of DNA encoding for viral protein (pCMV14-3X-Flag-SARS-CoV-2 S was a gift from Zhaohui Qian - Addgene plasmid # 145780; delta/beta spike expression plasmid kindly provided by Benjamin Murrell; Ebola GP expression plasmid kindly provided by Jochen Bodem), 7.5µg DNA encoding for HIV Vpr-GFP (NIH HIV Reagent Program, Division of AIDS, NIAID, NIH: pEGFP-Vpr, ARP-11386, contributed by Dr. Warner C. Greene), and 7.5µg encoding

for a lentiviral packaging plasmid (psPAX2 was a gift from Didier Trono - Addgene plasmid # 12260). Media was exchanged after 12 hours and VLPs harvested after 24 and 48 hours and enriched fiftyfold using LentiX concentrator according to the protocol provided by the manufacturer (Takara).

Microscopy and Quantification

Budding and labelling of VLPs was performed by co-expression of GAG (psPAX2) and its interaction partner Vpr-GFP, respectively. Labelling of the functional lipid bilayer covering fBSLBs was performed by addition of the membrane-integrating fluorescent lipid ASR-PE. After incubation with pseudotyped VLPs, fBSLBs were put into chambered glass coverslips (IBIDI: 81817) and imaging performed in PBS. Confocal microscopy was performed using a C-Apochromat 40x/1.20 water immersio objective of the Zeiss LSM780 microscope. Viral GFP was excited using 488nm argon laser and membrane-inserted ASR-PE was excited using a 633nm helium neon laser, while emission was collected from 498-552nm and 641-695nm, respectively. Full surface of 5µm fBSLBs was recorded by acquiring z-stacks with 24 slices each 0.3µm and VLP-GFP signal per bead quantified using ImageJ following the provided macro and automated workflow of Suppl. Fig. 02. To acquire fast, gentle, and big 3D volumes we used LLSM (Zeiss Lattice Lightsheet 7) with 488nm and 640nm laser excitation for viral GFP and ASR-PE, respectively. The general analysis workflow followed the one for confocal data, but parameters were adjusted for differences in signal intensity.

Flow Cytometry

Upon interaction of VLPs with fBSLBs the mixture was diluted in 500µl PBS and transferred into flow tubes. Flow cytometry was performed using a BD Fortessa system acquired at low speed and 488nm (FITC) or 640nm (APC) excitation/emission settings used for VLP-GFP and ASR-PE, respectively. 10 000 to 20 000 events were acquired and analysed using FCS Express 7 and Python (FCSParser). Gating was performed for all flow cytometry data as shown in Suppl. Fig. 04 on singlet bead population clearly visible in the forward- versus side-scatter plot. This population always represented the main bead population. Raw data of flow cytometry experiments is visualized as boxplots in Suppl. Fig. 05.

Serum Blocking

Human blood from healthy donors was obtained from blood transfusion station of Karolinska Hospital and serum prepared by centrifugation. The serum was aliquoted and frozen for further later use. To determine the amount of anti-spike IgGs in serum samples, $1x10^7$ 5µm silica beads (Bangs Laboratories) were washed three times with PBS and coated for 30 minutes with 47pmol SARS-CoV-2 RBD (BioSite: 40592-V08H)

on a rotary shaker (for this assay, beads are not coated with lipids since we mimic the ELISA assay with beads). After two washing steps with PBS beads were resuspended in 500µl PBS supplemented with 4mg/ml BSA to block non-specific interaction sites. 20µl of beads were incubated with stated serum dilutions over night at 4°C on a rotary shaker to enable interaction of anti-spike IgGs with coated beads. After two washing steps, anti-spike IgGs were labelled by incubation with 4µg/ml secondary anti-human IgG Alexa Fluor 488 antibodies (ThermoFischer: A11013) for one hour at room temperature on a rotary shaker in the dark. Labelled beads were washed and signal intensity of at least 9000 beads determined by flow cytometry. To test serum blocking efficiency, VLPs were pre-treated with stated serum concentrations over night at 4°C on a rotary shaker before incubated with ACE2-fBSLBs as described above.

Statistical Analysis

Visualization and statistical analysis of the data was performed using Python (Anaconda Navigator 2.3.2, JupyterLab 3.2.9) or GraphPad Prism. If data tested positive for normal distribution (Shapiro-Wilk test) an ordinary one-way ANOVA with multiple comparison test (Tukey) was applied, whereas for non-normal distributed data a Kruskal-Wallis H-test with post hoc pairwise test for multiple comparisons (Dunn's test with Bonferroni one-step correction) was applied. Figures were created with "SuperPlotsOfData" [4,5] to show the differences between biological replicates.

Supplementary Figures

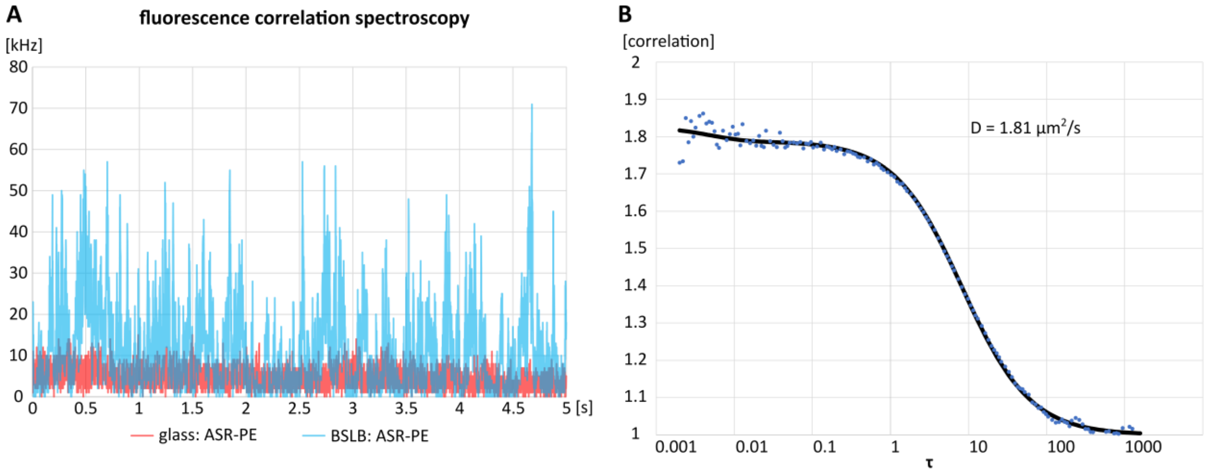

Suppl. Fig. 1: Proof of functional lipid-bilayer formation around 5µm silica beads. (A) Raw trace of FCS measurement of the lipid bilayer marker (ASR-PE) attached to 5µm silica beads in the presence (cyan) or absence (red) of the used lipid bilayer (98mol% POPC & 2mol% 18:1 DGS-Ni:NTA). Attachment and mobility of ASR-PE to the silica beads was only observed in the presence of a lipid bilayer (cyan), as derived from the fluorescence signal (y-axis: count rate) and fluctuations (x-axis: measurement time), respectively. **(B)** Diffusion coefficient of 1.81µm^2/s for ASR-PE in BSLB was obtained by fitting (black curve) of FCS-data (blue dots) with FoCuS-point software.

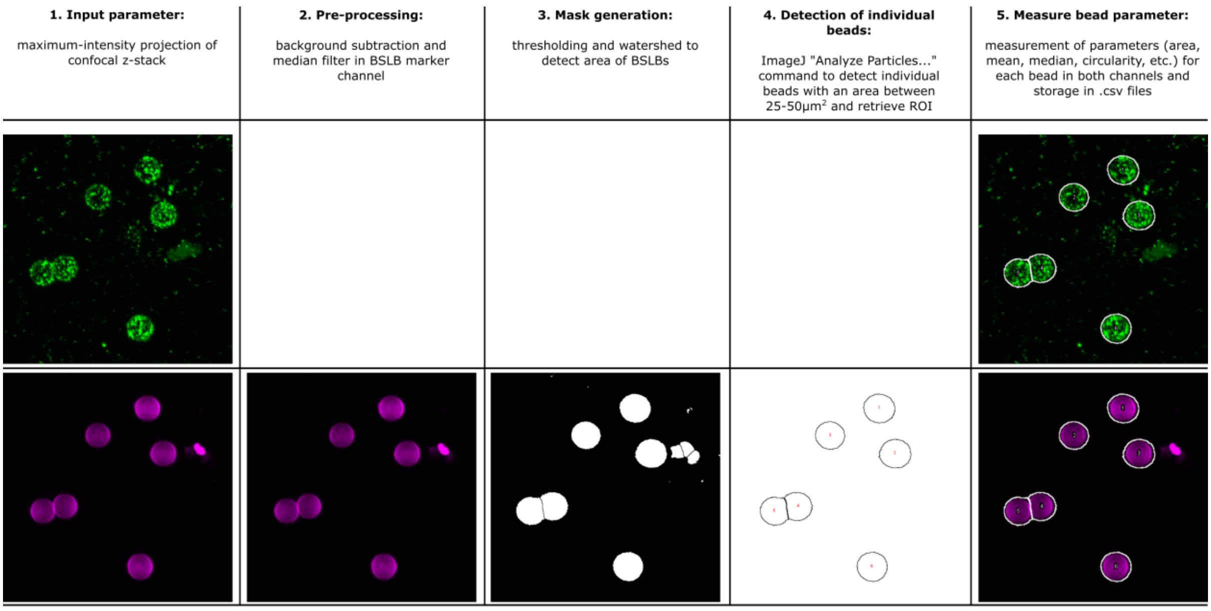

Suppl. Fig. 2: Automated image analysis workflow to determine VLP-GFP signal per fBSLB in confocal and LLSM data. Maximum-intensity projections of z-stacks were processed and thresholded to retrieve the region of interests (ROIs) of individual fBSLBs in the lipid-marker channel. ROIs were analyzed in the viral GFP-channel to extract mean and median intensity values per fBSLB.

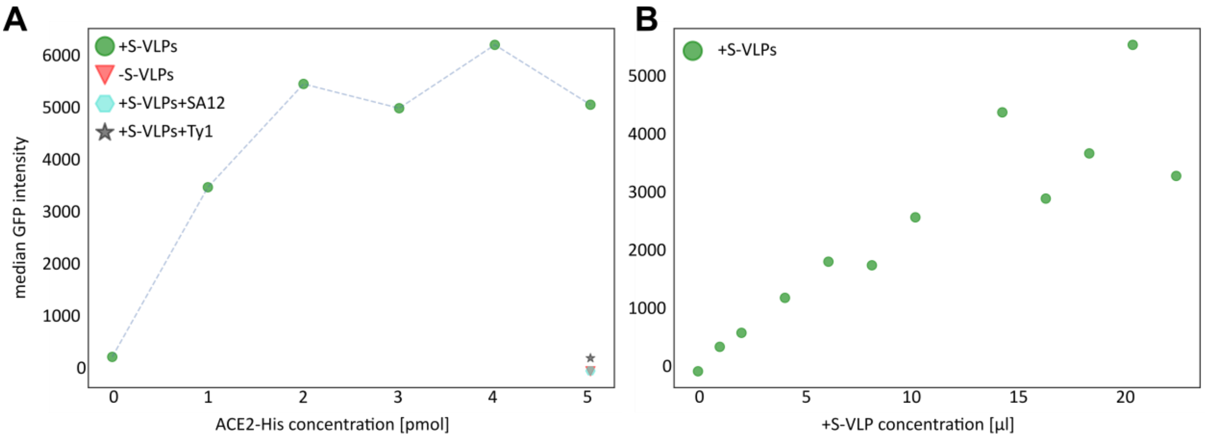

Suppl. Fig. 3: Optimal protein- and VLP-concentrations for ACE2-fBSLBs were determined by titration experiments combined with flow cytometry. (A) 10x10^6 BSLBs were functionalized with increasing amounts of ACE2-His protein and incubated with constant amount of Sp$^+$VLPs. Each dot in the scatterplot shows the median GFP-intensity of >20000 fBSLBs. **(B)** 10x10^6 BSLBs were functionalized with constant amount of ACE2-His protein and incubated with increasing concentrations of Sp$^+$VLPs. Each dot in the scatterplot shows the median GFP-intensity of >20000 fBSLBs.

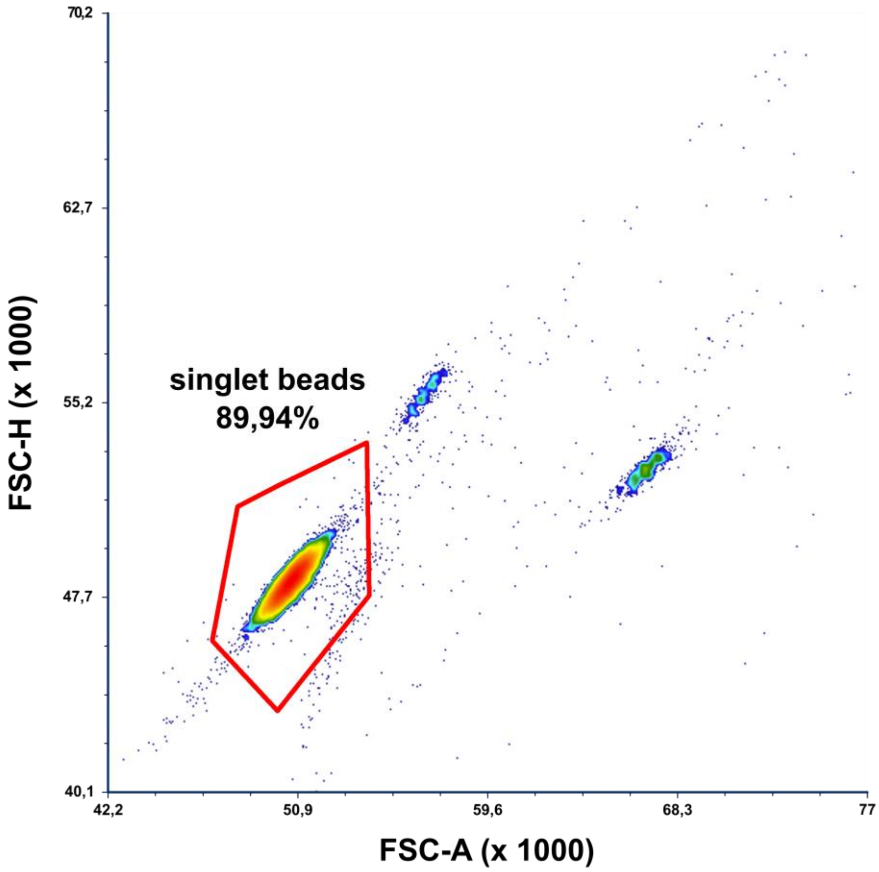

Suppl. Fig. 4: Flow cytometry gating strategy. Example density plot showing forward scatter area (FSC-A) plotted against forward scatter height (FSC-H) and the gated singlet bead population (red polygon).

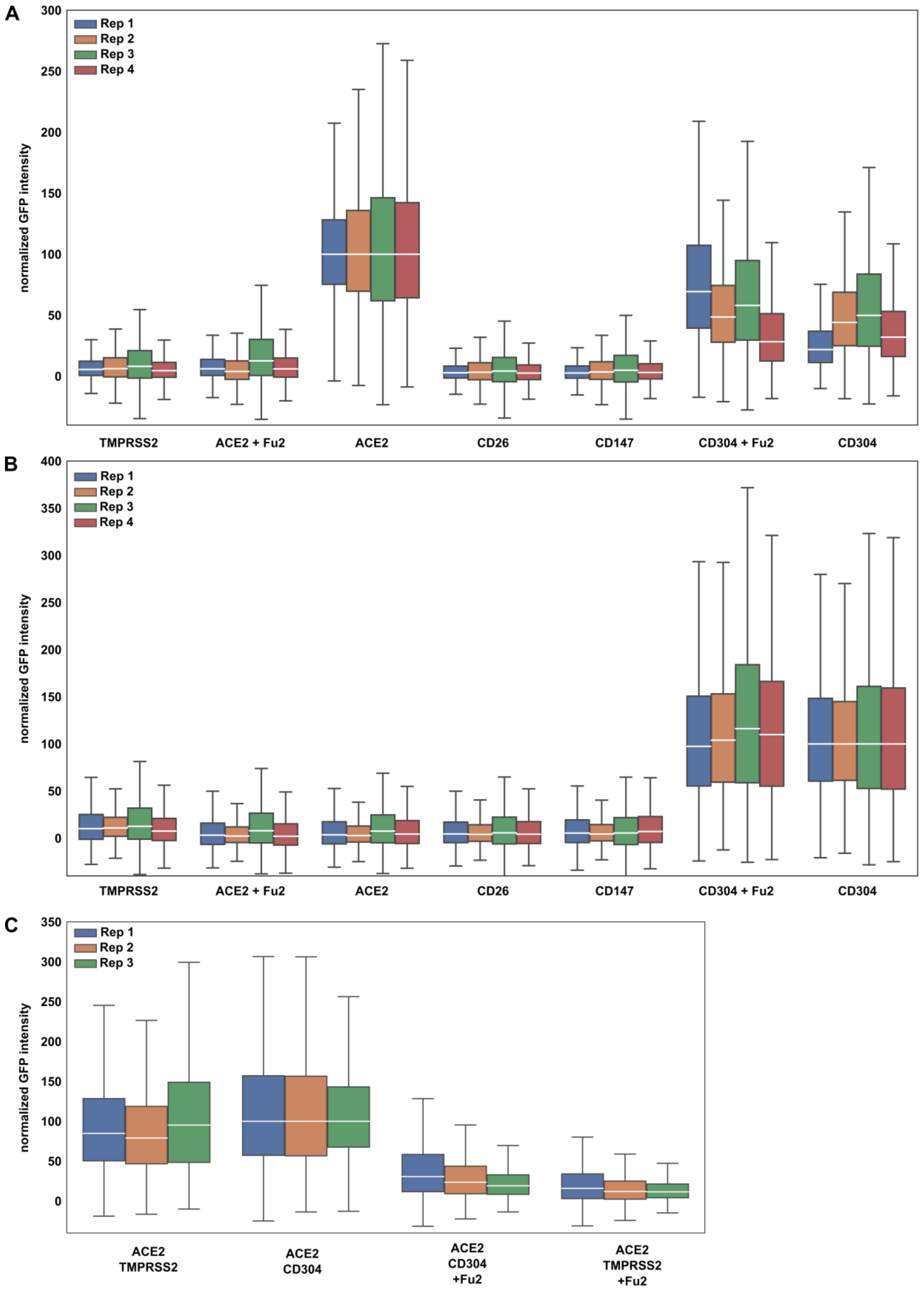

Suppl. Fig. 5: Box plots showing the corresponding median-normalized raw data distributions of (A,B) single- and (C) dual-receptor screens from main Figure 3. Box plots showing the quartiles of each data set with median line in white.

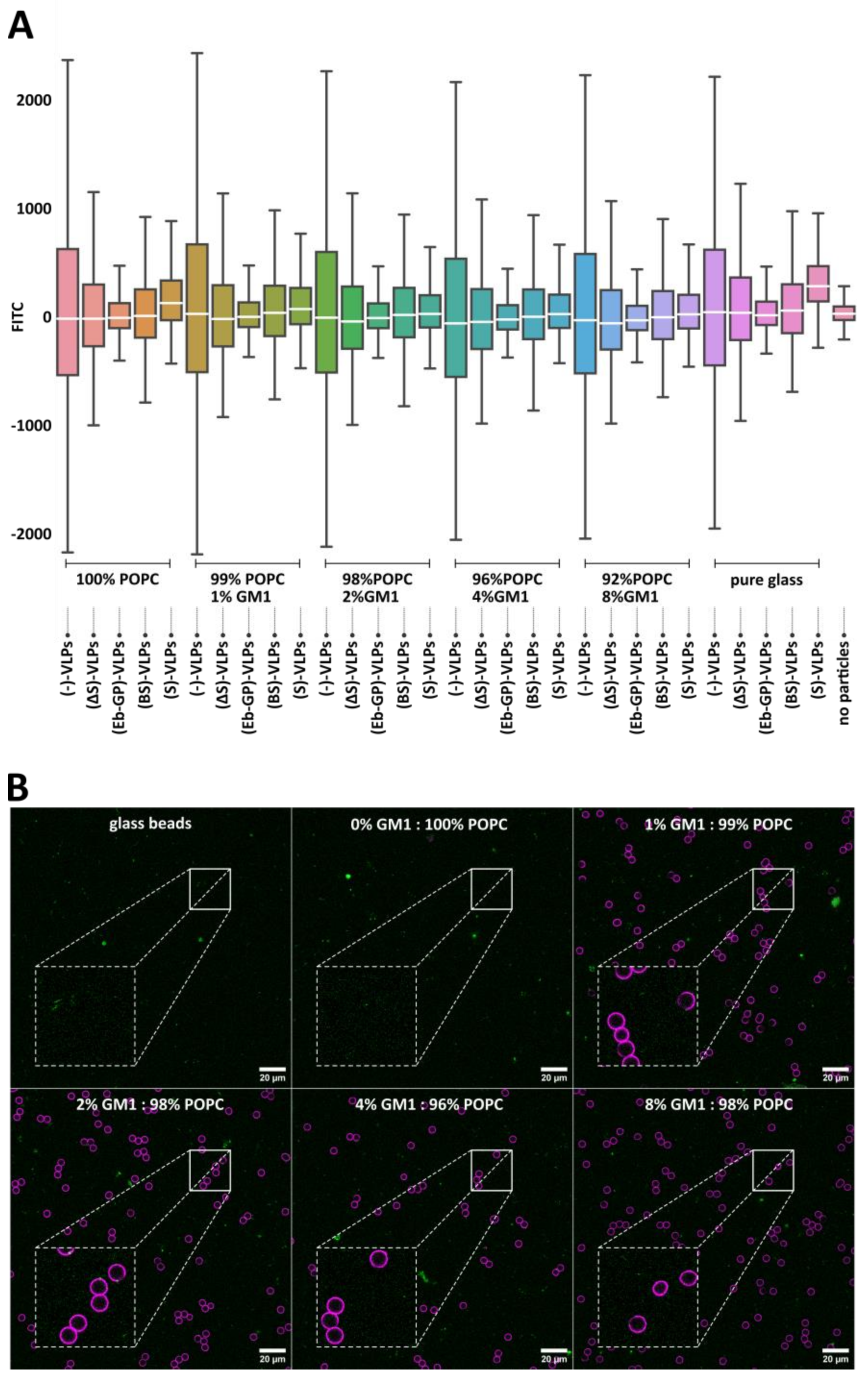

Suppl. Fig. 6: GM1-fBSLBs showed no increased binding to VLPs pseudotyped with different viral proteins. (A) Flow cytometry of VLPs pseudotyped with SARS-CoV-2 Spike (S) beta (B), delta (Δ), Ebola glycoprotein (GP) or no protein (-) to BSLBs coated with increasing GM1 concentrations showed no increased binding to GM1. Slightly increased binding was only observed for Sp^{+}VLPs to glass beads and POPC coated BSLBs. Box plots showing the quartiles of each data set with N ≥ 10000 with median line in white. **(B)** Concentration-dependent insertion of GM1 into fBSLBs was confirmed by cholera toxin B labelling (magenta) and confocal microscopy. Example showing GFP-tagged VLPs (green) pseudotyped with Δ-S.

Supplementary References

(1) Céspedes, P. F.; Jainarayanan, A.; Fernández-Messina, L.; Valvo, S.; Saliba, D. G.; Kurz, E.; Kvalvaag, A.; Chen, L.; Ganskow, C.; Colin-York, H.; Fritzsche, M.; Peng, Y.; Dong, T.; Johnson, E.; Siller-Farfán, J. A.; Dushek, O.; Sezgin, E.; Peacock, B.; Law, A.; Aubert, D.; Engledow, S.; Attar, M.; Hester, S.; Fischer, R.; Sánchez-Madrid, F.; Dustin, M. L. T-Cell Trans-Synaptic Vesicles Are Distinct and Carry Greater Effector Content than Constitutive Extracellular Vesicles. *Nat Commun* **2022**, *13* (1), 3460. https://doi.org/10.1038/s41467-022-31160-3.

(2) Hanke, L.; Vidakovics Perez, L.; Sheward, D. J.; Das, H.; Schulte, T.; Moliner-Morro, A.; Corcoran, M.; Achour, A.; Karlsson Hedestam, G. B.; Hällberg, B. M.; Murrell, B.; McInerney, G. M. An Alpaca Nanobody Neutralizes SARS-CoV-2 by Blocking Receptor Interaction. *Nat Commun* **2020**, *11* (1), 4420. https://doi.org/10.1038/s41467-020-18174-5.

(3) Hanke, L.; Das, H.; Sheward, D. J.; Perez Vidakovics, L.; Urgard, E.; Moliner-Morro, A.; Kim, C.; Karl, V.; Pankow, A.; Smith, N. L.; Porebski, B.; Fernandez-Capetillo, O.; Sezgin, E.; Pedersen, G. K.; Coquet, J. M.; Hällberg, B. M.; Murrell, B.; McInerney, G. M. A Bispecific Monomeric Nanobody Induces Spike Trimer Dimers and Neutralizes SARS-CoV-2 in Vivo. *Nat Commun* **2022**, *13* (1), 155. https://doi.org/10.1038/s41467-021-27610-z.

(4) Lord, S. J.; Velle, K. B.; Mullins, R. D.; Fritz-Laylin, L. K. SuperPlots: Communicating Reproducibility and Variability in Cell Biology. *Journal of Cell Biology* **2020**, *219* (6), e202001064. https://doi.org/10.1083/jcb.202001064.

(5) Goedhart, J. SuperPlotsOfData—a Web App for the Transparent Display and Quantitative Comparison of Continuous Data from Different Conditions. *MBoC* **2021**, *32* (6), 470–474. https://doi.org/10.1091/mbc.E20-09-0583.
